# Orbital floor fractures—a comparison between CT images and findings at surgery

**DOI:** 10.1007/s00405-022-07801-0

**Published:** 2023-01-10

**Authors:** Lena Folkestad, Lars Jönsson, Therese Karlsson

**Affiliations:** 1 ENT-Clinic, Södra Älvsborg Hospital, Borås, Sweden; 2 Department of Otolaryngology, Gothenburg University, 413 45 Gothenburg, Sweden; 3 Department of Radiology, Institute of Clinical Sciences, Sahlgrenska University Hospital, Gothenburg University, 413 45 Gothenburg, Sweden; 4 Department of Otorhinolaryngology, Head and Neck Surgery, Institute of Clinical Sciences, Sahlgrenska Academy, University of Gothenburg, Gothenburg, Sweden

**Keywords:** Orbital fracture, Computed tomography, Diplopia, Blow out fracture, Surgical findings, Concordance

## Abstract

**Purpose:**

The present study aims to investigate how well CT images correlate to surgical findings in orbital floor fractures and to the presence of diplopia.

**Methods:**

In this cross-sectional study, 27 consecutive patients already selected for surgery due to an orbital floor fracture underwent a routine CT scan (axial, coronal, sagittal). An ophthalmologist established any presence of diplopia. Extent of fracture/injury seen on CT was compared to that discovered during surgery.

**Results:**

In the surgeons´ opinions CT-images were in concordance with surgical findings in 71% of the cases. Agreement for pure blow out fractures was high (92%). Tetrapod fractures as a cause of an orbital floor fracture was only identified as such by surgeons in three of 11 cases, all subjected to orbital exploration, not only a closed reduction. Diplopia showed a significant correlation to rounding of the inferior rectus muscle at coronal CT. “Rounding” significantly correlated with the presence of a floor defect, to herniation of soft tissues and to the volume of displaced tissue.

**Conclusions:**

The results imply that the joint professional interaction between neuroradiology and surgery is important and would benefit from the use of an easy and well-defined classification system of orbital floor fractures. In Sweden a national record to collect data on all zygomaticomaxillary complex fractures assessed is to be started aiming at making general statements possible by time.

## Introduction

Orbital floor fractures as a concept are comprised of a variety of fractures that mainly can be divided into isolated blow out fractures and more or less comminuted fractures of the zygomatico maxillary complex. In addition, uncomplicated tetrapod fractures of the zygoma involve the orbital floor as defined by the anatomy, where the zygoma constitutes the lateral aspect of the orbital floor and maxillary bone constitutes the thin medial orbital floor. Consequently, a dislocated zygoma fracture usually affects the orbital floor to some extent. Most previous studies analyse pure blow out fractures as this is the most easily defined among the wide variety of fractures involving the orbital floor. However, what clinicians see in daily clinical work is a wide variety of zygomatico maxillary complex fractures, where orbital and ophthalmological effects are of central interest.

Treatment options are generally based on clinical signs and symptoms and the appearance of the fracture on CT (computed tomography) scans. Based on the available data in each individual case, it is finally up to the surgeon, in agreement with the patient to make the decision whether to operate or not. There are definite indications for surgery such as diplopia due to entrapment of the inferior rectus muscle and acute enophthalmos with or without a risk of developing diplopia. In the acute stage, soft tissue swelling often makes the clinical assessment more difficult to evaluate. Swelling due to hematoma or edema can cause restricted eye globe motility and also mask a present enophthalmos. To date, there is no definite consensus on the timing of surgery. On one hand, it is appropriate with early intervention before scarring and fibrosis commences. On the other hand, it is advisable to wait long enough for the swelling to subside to evaluate eye motility and globe position more reliable. Too early intervention, when swelling is still an issue, risks a compartment syndrome, endangering the optic nerve, which must be taken into account[1]. A general suggestion is to wait and see for 2 weeks, although some advocate an observation period of several weeks [2–6]. However, a trap door fracture of the orbital floor is an important exception, where muscle entrapment and/or a present oculo cardiac reflex are indications for immediate surgery within hours [6–8].

**Fig. 1:**
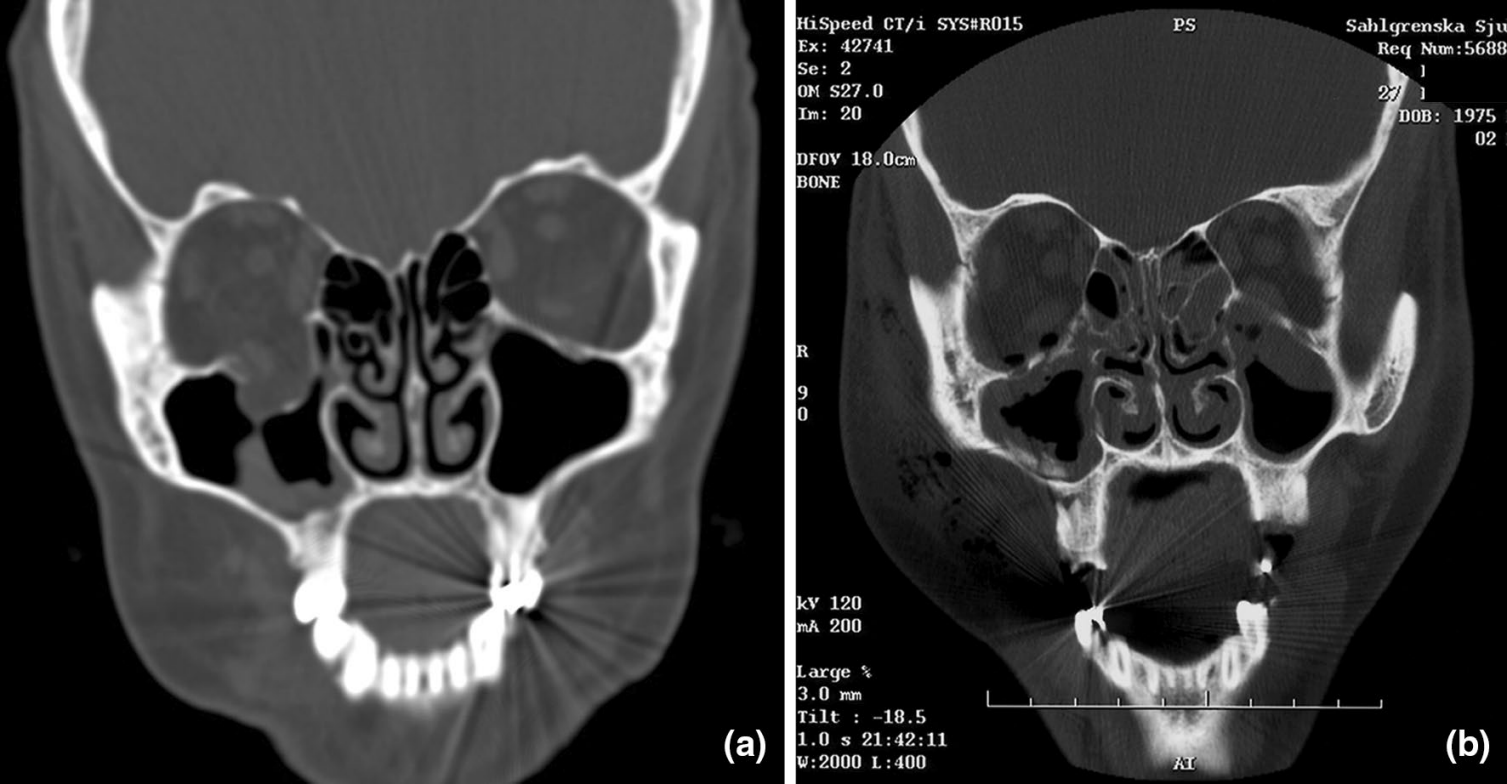
**a**, **b** Coronal CT scans. **a** Orbital floor defect with herniation of orbital soft tissues (right side), **b** “egg shell fracture” of orbital floor (right side)

Orbital floor fracture surgery comes with risk [9–11]. It is important to avoid surgery when it is not necessary. Beside the clinical assessment, reliable diagnostic tools are imperative. Computed Tomography scans (CT) and the forced duction test (fdt) give important contributions in diagnostics[8, 12]. CT (coronal, axial and sagittal views) usually gives substantial information regarding the injury. Several studies have established that a fracture size of 50% or more of the orbital floor and/or an orbital volume expansion exceeding 1.5–2 cm^3^, which has been shown to cause clinically obvious enophthalmos (≥ 2 mm), should be used as guidelines in the decision whether to operate or not [13–15]. However, a fracture comprising more than 50% of the floor can be anything from a large defect with herniation (Fig. 1a) to a floor fracture, where the fragments are kept together by periosteum/periorbita (“cracked egg shell”; Fig. 1b) the latter possibly with less risk of developing enophthalmos even if left without surgical intervention.

Not only are the bony structures of interest in orbital floor fractures but it has been shown that magnetic resonance tomography (MRT) can be used to discern entrapment of soft tissues in orbital fractures[16, 17]. However, to date the MRT is seldom available for this purpose in many clinics, but if so needs to be combined with a CT scan to provide information about the bony structures. Already in the 1980s it was suggested that oblique sagittal projections of the orbit gave valuable complements to the coronal CT views [18], especially in the assessment of the soft tissues, i.e., providing a possibility to evaluate the status of the inferior rectus muscle and its lengthwise extension [19].

**Fig. 2:**
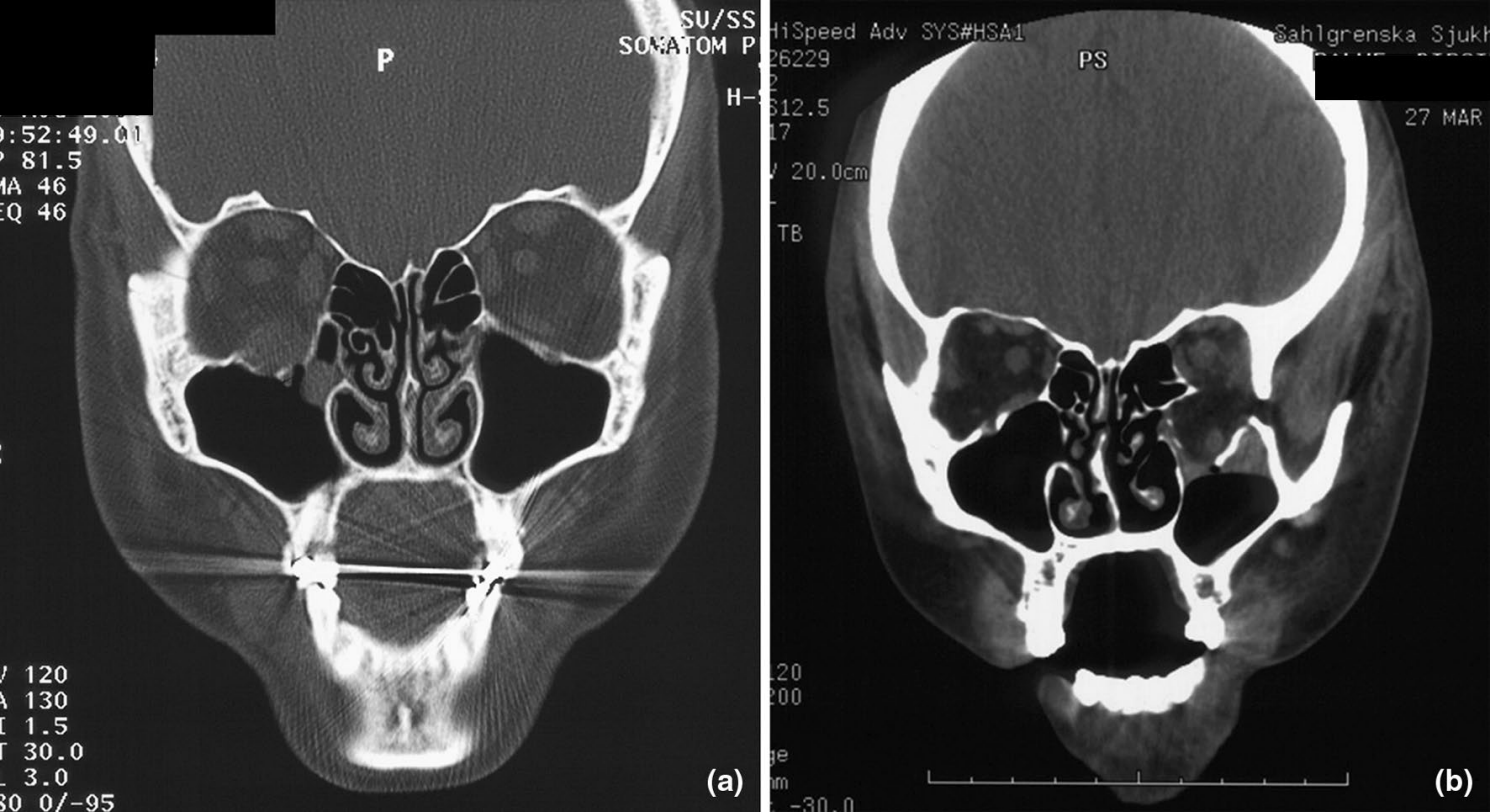
**a**, **b** “Rounding” of the inferior rectus muscle in coronal CT scans with an orbital floor fracture at the **a** right side, **b** left side, respectively

Rounding of the inferior rectus muscle at coronal CT images has been investigated in previous studies as a possible contributor to selecting surgery patients at risk of developing enophthalmos [20]. It was first described by Levine et al. in 1998 [21], in a case described with symptoms and signs in accordance with a trap door fracture. Rounding of the inferior rectus muscle is suggested to be caused by either edema; perimuscular or intramuscular haemorrhage; traction on the connective tissue network of the orbital soft tissues or; loss of eye globe support due to ruptured periorbit with herniation and loss of bony support (Fig. 2a, b) [16, 22, 23].

Despite guidance from experienced neuroradiologists, when it comes to interpreting the CT scans it seems to be a common reflection among surgeons that the extent of the fracture as revealed during surgery turns out to be larger than expected from the CT images. Hence, there is cause to compare CT with actual findings at surgery and ask for surgeons´ opinion on agreement in each case.

Previous studies of blow out fractures have shown that CT scans underestimate soft tissue herniation and entrapment when compared to surgical findings[21]. Since most studies only include blow out fractures, the present study aims at investigating the surgeons´ opinion of how well the preoperative CT scans correlate to the clinical and surgical findings in all orbital floor fractures and not only blow out fractures. Furthermore, the correlation of diplopia to findings at CT and surgery as well as how “rounding” of the inferior rectus muscle correlates to specific symptoms and signs were investigated.

## Materials and methods

### Study design

Twenty-seven consecutive patients who presented at Sahlgrenska University Hospital, Göteborg, Sweden, due to an orbital floor fracture during a time span of 18 months were included in the study. Inclusion criteria were a fracture involving the orbital floor verified at CT and surgical intervention considered necessary due to CT findings, clinical examination and symptoms.

The imaging studies were performed on a HiSpeed CT/i (GE Healthcare, Milwaukee, Wisconsin), 120 kV, 100 mA, SFOV 250 mm, DFOV 180 mm, slice thickness 3 mm and with bone algorithm. Some cases were performed at a neighboring hospital on a Somatom Plus (Siemens, Erlangen, Germany) with similar parameters. All CT scans (coronal, axial and reformatted sagittal) were evaluated and correlated with the preoperative clinical findings and the surgical findings.

Data on clinical symptoms and signs at the preoperative assessment were collected from the medical records for each patient. Prior to surgery, an ophthalmologist examined all patients.

To receive comparable data from CT images and intraoperative findings two separate study protocols were constructed besides the routine operative documentation of the operation for the medical record; Specific data concerning surgical findings was collected by means of a protocol filled in by the surgeon, whereas another specific study protocol was used to collect detailed information from the CT images by the neuroradiologist. Surgeons also used a Likert scale to grade the overall concordance between findings at surgery and CT images, as experienced from their point of view.

CT assessments for the present study were done retrospectively in a single blinded set up by an experienced neuroradiologist (L.J.) with no information about the patients or of findings at surgery. In addition, the surgeons could be considered blinded, as the purpose of the study protocol provided, was not known to them. As a test of intra-rater reliability, all CT images were evaluated twice, separated in time by at least 2 months and in an altered order to minimize the risk of the neuroradiologist recognizing individual images. The points of measurement used were defined by the orbital margin and the limits of the maxillary sinus roof, i.e., the orbital floor [13, 18]. All fractures diagnosed as blow out fractures were also assessed according to the method presented by Ploder et al. [13, 24].

### Ethical approval

The study was conducted in accordance with the Declaration of Helsinki. No ethical review was needed, because this is a quality evaluation of given care; thus, this does not fall under the Swedish law on ethical review of research. The study did not involve any patient contact nor did it affect treatment or patient outcome.

### Study variables and definitions

The variables studied emerge from Tables 1, 2, 3, 4, 5, 6, 7 and 8. Apart from “rounding” which could not be assessed at surgery, all findings established at CT scans and intraoperatively were compared and also when relevant, related to the presence of diplopia. Moreover, the surgeons opinion on concordance between preoperative CT findings and findings at surgery was asked for and graded in a Likert scale graded 1–5, where 1 meant the fracture was found to be much less extensive than expected, 3 meaning precise and as expected and 5 meant the fracture extensively exceeding as apprehended from the CT images.

**Table 1:** Descriptive data of the study cohort

Measures	Patients (*n* = 27)
Gender	
Male	21
Female	6
Age, median (years)	33 (range 15–68)
Side of injury	
Left	16
Right	11
Type of fracture	
Orbital floor in zygomatico-maxillary fracture	16
Blow out fracture	11
Causes	
Physical assault	10
Fall (high fall)	5 (2^a^)
Traffic accident (bicycle)	5 (3)
Sport	5
Accidentally hit	1
Missing value	1
Time to CT, median (days)	2 (range 0–13)
Time to surgery, median (days)	8 (range 5–18)
Orbital floor implant used	
None	3
Lyoplant^®^	14
Medpor^®^	9
Missing value	1

^a^One high fall occurred during sports activity

**Table 2:** Descriptive data of the blow out fractures (*n* = 11)

	Mean	Median	Range
Orbital floor area	6.1 cm^2^	5.9 cm^2^	5.1–8.0 cm^2^
Depth orbital cone	47 mm	45 mm	43–53 mm
Area of fracture (blow out)	2.7 cm^2^	2.8 cm^2^	1.1–4.0 cm^2^
Area % of floor	44%	51%	15–64%
VDT	1.4 cm^3^	0.99 cm^3^	0.15–3.6 cm^3^

**Table 3:** “Orbital floor fractures”—descriptive data of findings at CT scans and surgery

	CT	Surgery
Classification/Type of fracture		
Blow out	11	12
Orbital floor in zygomatico-maxillary	5	12
Tetrapod zygoma fracture	11	3
Area of fracture (% of floor);		
< 50%	17	22
50% ≤	10	4
Missing value	0	1
Floor defect	14	14
“Egg shell” fracture	11	8
Missing value	2	4
Herniation of soft tissue	12	13

**Table 4:** Concordance between CT images and findings at surgery/clinical examination

	Degree of agreement (%)	Strength of agreement (Cohen’s *κ*)
Type of fracture;		0.518 (*moderate*)
Blow out	92	
ZMC	80	
Tetrapod ZMC	27	
Size of fracture	80	0.431 (*moderate*)
Herniation of soft tissue	85	0.769 (*good*)
Possible entrapment^a^	100	0.489 (*moderate*)

^a^As assessed from CT scans and by ophthalmologist

**Table 5:** *Diplopia* in relation to co-existing variables

	Diplopia present (*n* = 10)	Diplopia not present (*n* = 16)	*p* value
Floor defect at CT	9	7	0.04
Herniation at surgery	8	5	0.04
Possible entrapment	8	6	0.05
VDT ≤ 0.5 cm^3^	2	11	0.01
VDT ≥ 1.5 cm^3^	4	1	0.01
“Rounding” of IRM	9	6	0.01

*IRM* inferior rectus muscle

**Table 6:** *Rounding* of the inferior rectus muscle in relation to co-existing variables

	“Rounding” present (*n* = 14)	“Rounding” not present (*n* = 13)	*p* value
Floor defect at CT	11	4	0.057
Herniation at surgery	10	3	0.047
Possible entrapment at CT;	9	5	0.26 no s
Forced duction test Positive (*n* = 1)	1	0	
Negative (*n* = 5)	1	4	
neg after reduction (*n* = 9)	7	2	
Periorbit ruptured at CT	11	4	0.057
VDT ≤ 0.5 cm^3^ at CT	5	9	0.019
VDT ≥ 1.5 cm^3^ at CT	5	0	0.019

**Table 7:** Descriptive data of diplopia and result of forced duction test (FDT) at surgery

	Diplopia present (*n* = 10)	Diplopia not present (*n* = 16)
FDT positive	1	0
FDT negative	2	3
FDT negative after reduction	4	5
No FDT performed	3	8

Missing value:1

**Table 8:** *Eye globe position* as assessed by CT in relation to variables

	Enophthalmos (*n* = 2)	Exophthalmos (*n* = 12)	Normal (*n* = 12)	*p* value
Herniation at surgery	2	5	6	no s
VDT ≤ 0.5 cm^3^	0	6	7	0.14
VDT ≥ 1.5 cm^3^	2	1	2	0.14
“Rounding” of IRM	2	5	8	no s
Diplopia	2	4	4	no s

Missing value: 1

*IRM* inferior rectus muscle

Herniation was defined as the presence of orbital soft tissue in the maxillary sinus, i.e., in between and beneath the bone fragments of the orbital floor fracture. Using this definition herniation could only be present when there was a floor defect and not in cases of so called “egg shell fractures” of the orbital floor. On CT images herniation and/or intraorbital air were judged to be signs of a ruptured periorbit. Volume of displaced tissue (VDT) was defined as to include both cases of herniation as well as “egg-shell fractures” extending below normal floor position, with a subsequent increase of the orbital volume. Rounding of the inferior rectus muscle was established as present when the muscle at the injured side appeared round at coronal views in contrast to the normal flattened shape at the uninjured side. When, on the CT scans, bony fragments or any edges of the fracture were considered to possibly compromise the function of the inferior rectus muscle, this was assessed as a potential for tethering/entrapment. If such findings, in patients with diplopia, were also established at surgery, as well as if the forced duction test was positive, this was considered to verify entrapment.

### Statistics

Cohen´s kappa was used to assess intra-rater reliability (neuroradiologist) and concordance between fracture characteristics appearing on CT scans and at surgery.

The significance of correlations between variables was calculated with Fisher´s exact test. Categorized data were calculated using Mantel–Haenzel test. The statistical significance was set on *α* = 0.05. All statistics were performed with the SPSS for Windows, version 22.0, software.

## Results

The computed tomography (CT) scans of 27 consecutive patients with an acute orbital floor fracture, 11 of which had a pure blow out fracture, were studied and compared to findings at surgery. The intra-rater reliability for the neuroradiologist´s assessment of the CT scans, separated in time by at least 2 months, was “very good” (*κ* = 1).

Descriptive data are accounted for in Table 1. The male-to-female ratio was 3,5:1. The median age of the men was 33 years (range 15–59 years) and of the women 49 years (range 17–68 years). Ten patients had preoperative diplopia, seven of which had a blowout fracture. All cases of diplopia were confirmed by an ophthalmologist.

In general, 6 days (range 1–15 days) had passed between the CT scan and surgery. Two patients, both with a blowout fracture, had late surgery, i.e., more than 2 weeks after the trauma (17 and 18 days). Ten different surgeons had carried out the operations.

In all 27 cases, the neuroradiologist established from the CT scans whether the fracture involved more or less than 25% or 50% of the orbital floor area. The distribution of cases within these size spans proved to be equal (< 25% = 9; 25–49% = 8; ≥ 50% = 10). The blow out fractures (*n* = 11) were also measured according to the method of Ploder et al. (Table 2). The mean fracture area among the blow out fractures was 2.7 cm^2^ corresponding to 44% of the orbital floor.

### Concordance between findings on CT and at surgery

The agreement of CT scans with intraoperative findings is shown in Tables 3 and 4. Interestingly, 11 of the orbital floor fractures selected for open surgery proved to be straightforward tetrapod fractures of the zygoma. The surgeon established the diagnosis tetrapod fracture of the zygoma in only three cases of 11. In five of these (45%) the orbital floor fracture became reduced to satisfaction already by means of a Gillie´s procedure and no stable orbital floor implant was required. As a result of difficulties to achieve a sufficient Gillie´s reduction, another three cases (3/11) resulted in an incongruent orbital floor and a 0.85 mm sheet of Medpor^®^ was used to fill the void space.

Less than half the number of floor fractures comprising 50% or more of the floor area as assessed from CT scans (10) were recognized as that large during surgery (4) (Table 3). The most consistent type of fracture, the most easily defined, were the pure blow out fractures (Tables 3 and 4). It only differed in one case, where an orbital floor fracture with a non-dislocated fracture of the inferior orbital rim and fractures of the lateral maxillary sinus wall were classified as a blowout fracture from the surgeon´s point of view. Regarding the presence of a floor defect rather than an “egg shell fracture” as well as coinciding presence of herniation of orbital soft tissue, there was generally a high concordance between findings on CT images and at surgery (Table 3).

According to the Likert scales, surgeons estimated the overall agreement between preoperative CT scans and findings at surgery to be good (Likert = 3) in 15 of 21 cases (71%). The fracture was considered more extensive (Likert = 4) than expected from CT scans in four cases (three tetrapod fractures, one blow out fracture) and less/considerably less (Likert = 1 and 2) than expected in two (one tetrapod fracture, one zygomatico maxillary//orbital fracture).

### CT findings and diplopia

A floor defect, herniation and/or signs of possible entrapment were likely to be seen at CT images when a patient had diplopia (Table 5). Diplopia was present regardless of whether fractures were small or large. There was a statistically significant correlation between diplopia and “rounding” of the inferior rectus muscle at coronal CT-scans (Table 5). “Rounding” of the inferior rectus muscle is an image characteristic that cannot be estimated at surgery. Instead “rounding” on CT was compared to the presence of herniation of soft tissue at surgery with a subsequent possibility of muscle kinking or rupture of the periorbit, and to a positive forced duction test if caused by tethering of the muscle or adjacent septae of the ligamentus apparatus (Table 6). “Rounding” was present in 52% (14/27) (Table 6). All patients with restricted eye motility (6/24: 25%) showed a “rounded” inferior rectus muscle at the injured side on coronal CT scans. “Rounding” showed a significant correlation to a floor defect with herniation at surgery, to the presence of a floor defect and to signs of a ruptured periorbit at CT. Whether the volume of displaced tissue was less than 0.5 cm3 or larger than 1.5 cm3, there was a significant correlation with rounding. This might be explained by different causes for the “rounding” as suggested previously such as kinking of the muscle in a large herniation or tethering of orbital septae in small or linear floor fractures, both of which can also explain restricted eye motility and diplopia.

Forced duction tests (FDT) were conducted in too few patients for conclusions to be drawn (Table 7). No FDT was performed prior to surgery in topical anesthesia in the awake patient. No FDT was performed at the start of the operation if the patients did not suffer from preoperative diplopia. In cases of diplopia, FDT was done at the start of the operation in approximately one-third of the patients, following reduction in one-third and not at all in one-third. Only one case of restricted eye motility was verified by a positive FDT. In the remaining, patients, FDT was used only to verify free motility after reduction of the fracture and insertion of an orbital floor implant.

### CT findings and eye globe position

As established from the axial CT scans, eye globe position was unaffected in 13/27 cases (48%, Table 8) and exophthalmos was established in 12 patients (12/27, 44%). Enophthalmos was seen in two (8%). Exophthalmos was present in six (6/11, 54%) (intraindividual difference in globe position: 2–6 mm) and enophthalmos was seen in two of the blow out fractures (2/11, 18%). In the remaining three cases of blow out fractures, the bulb position was unaffected despite a fracture area being larger than 50% of the floor and volume of displaced tissue exceeding 1.5 cm^3^.

There were no major differences as to when CT scans were conducted in relation to the established eye globe position. Patients showing exophthalmos had in median the CT scan executed on day 1 after the trauma, patients with a normal bulb position on day 2. The two patients with enophthalmos had their CT scan done on days 0 and 2, respectively. Both cases of enophthalmos were established as such by the ophthalmologist. However, exophthalmos was registered in only one of the two most severe cases (difference of 5 and 6 mm, respectively).

## Discussion

Most studies of orbital floor fractures are confined to studies on *blow out fractures, i.e., isolated or pure orbital floor fractures*. However, *orbital floor fractures* in daily practice comprise so many more variants than only the pure blow out fractures of the orbit. In a previous retrospective study of orbital floor fractures less than half (44%) of 107 patients had a pure blow out fracture [11]. Thereby, it is of interest to investigate which fractures are accounted for as “orbital floor fractures” in daily practice and how they are treated.

Descriptive data from the present study are in accordance with previous studies and thus supports that the studied group of patients are representative of orbital floor fracture patients. The availability of sagittal reconstructions and today’s 3D imaging have possibly enhanced and increased surgeons’ understanding of different orbital floor fracture features. Dubois et al. have recently showed that CT measurements are the most consistent and accurate tool for estimating the size of orbital fractures [25]. On the other hand, Vicinanzo et al. emphasize the importance of basing the decision whether to operate or not on the clinical findings as the variability in CT assessment even for experienced neuroradiologists is significant and troublesome [25]. Recent anatomical studies of human orbits have shown that both orbital volume and eye globe volume and the relation between the two may differ considerably between individuals [26]. This should be kept in mind when applying the widely accepted guideline that orbital floor fractures exceeding 50% of the floor and an orbital volume increased by 1.5 cm^3^ or more should indicate surgical intervention. A fracture area of 50% of the floor without herniation is no definite indication for surgery. In the present study none of the fractures assessed as “egg shell” fractures had a volume of displaced tissue exceeding 0.6 cm3. There are reasons to believe that even large “egg shell” fractures have a potential of spontaneous healing without troublesome sequelae [20].

Surprisingly, and in contrast with the common impression that surgeons often reckon the fracture larger during surgery than expected from the preoperative CT images, in the present study less than half the number of the large floor fractures (50% or more of the floor area) as assessed from CT scans (10) were recognized as that large during surgery (4). This difference could be explained by surgeons tending to estimate the size of the defect only, while on CT scans the entire fractured area was included also counting for areas with minimally dislocated bone fragments. Interestingly, 41% (11/27) of the “orbital floor fractures” selected for open surgery (orbital floor exploration) in the present study were uncomplicated tetrapod fractures of the zygoma. Tetrapod fractures were obvious to the neuroradiologist. However, although the fracture lines were described in the CT report, eight out of the 11 were not recognized as such by the surgeons. Instead they were looked upon as “orbital floor fractures” and subsequently subjected to open surgery via a subciliary approach. In none of these tetrapod fractures was a stable floor implant needed to cover a defect. Medpor was used to fill the void in three cases, where reduction according to Gillie’s was difficult, most likely due to late surgical intervention (9 days or more after injury).

The tetrapod fractures were also responsible for the main part of discrepancies in how well the CT images agreed with findings at surgery according to surgeons´ opinion, graded by the Likert scales. What had been classified as a straightforward tetrapod fracture of the zygoma at CT scans, had to a large extent by the surgeon, been classified as an orbital floor fracture and as such subjected to open orbital exploration. The fact that the surgeons and not the neuroradiologist, make the decision whether to operate or not, may, therefore, explain why so many tetrapod zygoma fractures were subjected to open surgery as “orbital floor fractures”. This highlights a shortcoming in communication and lacking the use of an easy, clear and well-defined classification system as a tool to enhance the professional interaction between neuroradiologist and surgeon when it comes to “orbital floor fractures”. If the tetrapod fractures of the present study had been as obvious to the surgeon as to the neuroradiologist, the number of orbital explorations may have been reduced by 41% (11/27).

There are a few classification systems suggested for “pure orbital floor fractures”, i.e., blow out fractures [27–30], but for the wider concept of “orbital floor fractures” in zygomaticomaxillary complex fractures, this is more complicated to achieve. The three-level craniomaxillofacial classification developed by the AOCMF Classification Group [31] is based on involvement of the bony structures and depicts orbital fractures according to the subregions defined as orbital rims, anterior orbital walls, midorbit and apex and on a more detailed level also specific orbital structures such as the inferior orbital fissure, the internal orbital buttress, greater wing of sphenoid, lacrimal bone, superior orbital fissure and optic canal. However, any assessment of the orbital soft tissue content is not included in this classification but is of important clinical, therapeutic relevance. As an example, in the present study, a clear significant correlation (*p* = 0.01) was established between rounding of the inferior rectus muscle and diplopia.

The relatively clear and well-defined concept of blow out fracture, the pure/isolated orbital floor fracture, probably explains the high concordance for these in the present study. Our suggestion, based on the results of the present study, is that not only blow out fractures but also tetrapod zygoma fractures should be clearly distinguished from other fractures involving the orbital floor, as the surgical approach to the latter often can be confined to closed reduction [5, 31, 32]. Simply using this terminology in the CT-report can increase the awareness of this among surgeons.

Apart from classification of the fracture, calculation of the fracture size, the volume of displaced soft tissue and the presence of any herniation, additional information from CT-imaging such as distinguishing a defect from an “egg shell” fracture, notifying the presence of intraorbital air as a sign of a ruptured periorbit and rounding of the inferior rectus muscle, could routinely be commented on the CT-report according to a check list. This would provide a foundation for a structural and straightforward professional joint discussion and, by time an increasing body of evidence and knowledge not only in assessing the bony structures but also when it comes to evaluating signs reflecting the condition of the orbital soft tissues.

Preferably, a straightforward tetrapod fracture should be subjected to a closed reduction and a forced duction test performed before as well as after reduction to ensure no soft tissue becoming impinged during manipulation of the fracture. In cases of instability, the fracture can often be stabilized at the buttress by rigid fixation via an oral approach, with no subsequent risk of developing a visible scar [31, 32]. The use of intraoperative CT, suggested by Manson [32] already in 1999 has further improved the chances of evaluating the result of reduction already during surgery, and also provide clues to if open orbital floor surgery is required and thus performed only when necessary.

In Sweden, a national record is to be started aimed at increasing the body of evidence on orbital fractures, by collecting data on all fractures involving the orbit assessed and over time, making general statements on orbital fractures and “orbital floor fractures” in particular, possible. A similar opinion has recently been put forward by Dubois et al. [33]. They suggest uniform standardized tests, validated questionnaires in combination with three-dimensional volume-based defect classification, should be used in orbital fracture research in the coming decade.

The strengths of this study lie in the high intra-rater reliability, blinding aspects as well as high consistency due to all CT images being evaluated by the same neuroradiologist. It is, however, limited by the small number of cases albeit these still appear representative of the fracture types seen in patients with orbital floor fractures. In addition, as many as ten different surgeons operated the 27 patients—all with different experiences, which should be taken into account as a more experienced surgeon may classify a fracture differently compared to a less experienced one.

## Conclusions

It is clear that findings reported by neuroradiologists (based on CT scans) and surgeons differ regarding orbital floor fractures. The results from this study highlight that the routine use of a well-defined classification system of orbital floor fractures and a standardized CT reporting protocol would greatly enhance professional communication between neuroradiologists and surgeons, thereby avoiding unnecessary orbital surgery.
